# Working conditions of healthcare workers and clients’ satisfaction with care: study protocol and baseline results of a cluster-randomised workplace intervention

**DOI:** 10.1186/s12889-020-09290-4

**Published:** 2020-08-25

**Authors:** Diego Montano, Marco Kuchenbaur, Heinrich Geissler, Richard Peter

**Affiliations:** 1 Department of Medical Sociology, Faculty of Medicine, Institute of the History, Philosophy and Ethics of Medicine, Ulm University, Parkstr. 11, 89073 Ulm, Germany; 2 Arbeit und Zukunft e.V, Hamburg, Germany

**Keywords:** Work ability, Work stress, Effort-reward imbalance, Organisational interventions, Psychosocial load

## Abstract

**Background:**

In the present investigation the study protocol and the results at baseline of a workplace intervention are reported. It is hypothesised that the reduction of the physical and psychosocial workload of healthcare workers increases 1 their self-assessed physical and mental work ability, and 2. clients’ satisfaction with care.

**Methods:**

Two-arm, cluster-randomised trial. Outcome data on workers and clients are collected in questionnaires at baseline, and two follow-ups between 2019 and 2021. Participants of the interventions are healthcare workers of 11 healthcare providers in Germany. At baseline, the intervention arm comprised 22 clusters (*n* = 174 workers); the control arm, 47 clusters (*n* = 276). The intervention consists of interviews and workshops, in which employees propose measures aiming to reduce the physical and psychosocial load, and strengthen resources at work. The primary outcome is the workers’ physical and mental work ability. The secondary outcome is the clients’ satisfaction with care.

**Results:**

There was no evidence of substantial differences between trial arms at baseline concerning the outcomes. The design effect estimates for physical and mental work ability were 1.29 and 1.05, respectively. At the end of the trial, effect sizes of at least 0.30 and 0.27 at the 80% power and 5% significance levels can be attained.

**Conclusions:**

The results suggest that the implementation of the study design has been satisfactory. The intervention is expected to provide evidence of relatively small to medium-size effects of the intervention activities on the work ability of healthcare workers and the clients’ satisfaction with care.

**Trial registration:**

Registration trial DRKS00021138 on the German Registry of Clinical Studies (DRKS), retrospectively registered on 25 March, 2020.

## Background

In Europe, nurses’ intention to give up their profession have been found to increase with the perception of higher job-related efforts, lower rewards at work, and stronger overcommitment to job demands [1]. Meta-analytic results have indicated that psychosocial factors involving high job demands and low job control are associated with prevalent and incident musculoskeletal symptoms involving neck, shoulder and back pain among hospital nurses and nursing aides [2]. In addition, the so-called effort-reward imbalance [3], i.e., the combination of high efforts and low rewards obtained from one’s work, has been related to larger odds ratios for the experience of burnout symptoms among nurses, particularly in Germany where higher levels of effort-reward imbalance at work have been reported [4]. A previous systematic review by Duhoux et al. (2017) on non-randomised workplace interventions aiming to promote the mental health of primary care nurses, revealed that burnout and job stress could be reduced by different types of person-centred interventions including cognitive behavioural and mindfulness techniques [5]. However, despite that organisational interventions are regarded as more effective strategies of health risk prevention, only one organisational intervention met the inclusion criteria in that systematic review. Furthermore, no randomised controlled interventions were included, even though the risk of bias in complex intervention studies can substantially be reduced by randomisation [6]. In the large meta-analysis by Ruotsalainen et al. (2015) on workplace interventions aiming to reduce the perceived job stress in healthcare workers, it was found that two organisational interventions comparing an intensive participatory programme for the improvement of working conditions to no intervention were not effective at reducing the workers’ job stress levels [7]. In addition, according to the results reported by Ruotsalainen et al. (2015) participative, randomised workplace interventions with a follow-up time of more than 12 months were also extremely scarce: From the 21 organisational interventions found by the authors, only the study of Uchiyama et al. (2013) was a cluster-randomised controlled participatory intervention [8], whose effects, however, were measured immediately after the six-month intervention and, consequently, are of limited validity. Thus, to the knowledge of the authors and the literature aforementioned, the effectiveness of participatory organisational interventions in healthcare workers at the workplace has been barely investigated within the methodological framework of randomised controlled trials.

Hence, the present study contributes to research by presenting the study protocol and baseline results of a cluster-randomised workplace intervention among healthcare workers: “HALTgeben” (“Higher Patient Satisfaction through Fair Working Conditions in Healthcare”). The study HALTgeben is an organisational workplace intervention with healthcare workers conducted in 11 German health services providers which aims to improve the work ability of workers, and, thereby, the satisfaction with care of hospital patients and individuals in elderly care (i.e. the clients). It is hypothesised that the reduction of physical and psychosocial workload of workers increases 1. their self-assessed physical and mental work ability, and 2. clients’ satisfaction with care. These hypotheses pertain the individual level (the self-assessment of work ability and clients’ satisfaction with care), and the cluster level as well (average workload of healthcare workers in the clusters). In the following sections, the study protocol and the results at baseline are reported according to the CONSORT Statement for cluster-randomised trials (see also the Supplementary material 2 for the corresponding check-list) [9].

## Methods

### Study design

The study is a two-arm, cluster-randomised intervention with healthcare workers conducted in seven general and three specialised hospitals, and an elderly care centre in Germany, whose wards constitute the clusters. A cluster-randomisation design was required due to the fact that it is an organisational workplace intervention whose main target are wards, and the randomisation of individuals is not feasible, since, in principle, the set of measures implemented in the intervention wards may affect all workers therein. A cluster-randomisation design helps reducing the risk of contamination effects between intervention and control wards and, at the same time, accounts for the correlations of individual measurements being observed within clusters. Outcome data are collected at baseline and at two follow-up times (T1 and T2) in surveys containing validated instruments. Data collection at baseline was performed before the interventions began in the intervention arm. The first follow-up measurement T1 in the single wards will be conducted successively no later than 6 weeks after the implementation of the first measures aiming to reduce the workload of healthcare workers. The final measurement T2 in all wards will be performed 12 months after the last T1 follow-up. Baseline data collection on workers and clients took place between June and December 2019. The follow-up measurements at T1 and T2 will take place in 2020 and 2021, respectively.

### Participants

#### Healthcare workers

Eligibility criteria of individuals to participate in the workers survey were being employed as a healthcare worker, being older than 18 years, and working most of the time in a single ward only. A census of the healthcare workers population was attempted, and, therefore, all eligible workers were contacted by mail and invited to participate in the surveys. Each contacted employee received a booklet with a brief description of the study, data privacy policies for the surveys, a registration sheet, and the corresponding consent form. Participants were asked to provide written informed consent prior to study enrolment and supply the name of the ward they usually work in. The information on wards was validated with internal lists provided by the hospitals and the elderly care centre. Hence, the clusters were defined based on the information supplied by the workers, the internal organisational structure and the type of health services provided in the healthcare organisations. Eligibility criterion for clusters was the unambiguous assignment to patient or elderly care, respectively. The enrolment of healthcare workers ended on 31 October, 2019.

#### Clients

The eligibility criteria for participation in the client surveys were being older than 18 years, being able to give informed consent to participation, being a responsive patient, and having sufficient skills in the German language. Clients (i.e. patients and individuals in elderly care in residencies and at home) are contacted in the participating healthcare organisations and receive a booklet with a brief description of the survey and data privacy information. All clients are required to consent explicitly to participate in the study before data collection. At baseline, T1 and T2 follow-up, approximately 600 patients and 150 individuals in elderly care will be surveyed successively in a cross-sectional design in the intervention and control wards. Participation in the client surveys is anonymous. The outcome data on clients are collected by interviewers either as self-administered questionnaires, or personal interviews upon clients’ request. The interviewers receive a three-hour training in survey methodology provided by the authors from Ulm University before data collection.

### The intervention

**Fig. 1:**
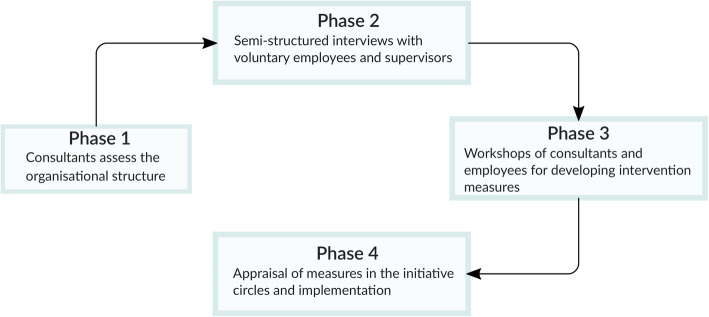
Schematic representation of the intervention phases

The intervention addresses healthcare workers only, and is performed by four consultants whose areas of expertise cover work design and organisational development. Even though the interventions target whole wards in the intervention arm, data on workload and work ability is available only from employees consenting to participate in the worker surveys (i.e., not all workers in the single intervention wards take part in the surveys). The intervention is based on the concept of work ability [10, 11] and focuses on the balance between the individuals’ capacities and their work demands [12]. The main target of the intervention is to achieve that workers accomplish their work duties, by considering how individual characteristics and capacities of the individual workers may be aligned with the definition of work and task processes throughout different life phases. The consultants’ approach specifies four age-dependent main career stages: entrance, development and transition, continuity, and exit [12]. It is assumed that each career stage requires appropriate task and work specifications. The intervention is implemented in four phases (Fig. 1). Phase 1: The consultants ask the participating organisations for information regarding the organisation as a whole such as main work tasks of targeted employee groups, shift schedules, reports on occupational risk assessments, age structure, work council agreements, and work and operating instructions. Phase 2: Voluntary employees and supervisors in the intervention wards are interviewed and asked, among others, for their assessment on workplace aspects such as work organisation and processes, age-critical work tasks and workload, psychosocial demands, and degree of cooperation with colleagues of different ages. The interviews are conducted by means of a semi-structured questionnaire with an open-answer format. Phase 3: The consultants summarise the information provided by the organisations and the interviewed workers and supervisors according to the career stages mentioned above, and the five components of the work ability concept, namely, health capacity, occupational competence, attitudes and motivation, work organisation and management, and life-domain balance [11]. Afterwards, the interviewed workers are invited to participate in a workshop which lasts about 3 h. The consultants present and discuss the results in the workshop, and ask participants to propose measures aiming to enhance their work ability, improve their working conditions, and adapt the work environment to an ageing workforce. Phase 4: In each participating organisation so-called “initiatives circles” are implemented, in which the intervention measures proposed in the workshops are appraised regarding their feasibility. Members of the initiative circles may be managing board executives, managers of the healthcare departments, works council, and quality management or human resources representatives, who decide which measures can be implemented by the intervention wards themselves, and which require executive board approval. From a temporal perspective, the measures are categorised as short-term (e.g., ergonomic measures), medium-term (e.g., alignment of shift schedules according to workers’ needs in the different life phases), and long-term (e.g., personnel recruitment, work processes between departments or occupations). From a content perspective, the measures are categorised as individual (e.g., exercise programmes), inter-personal (e.g., health-promoting leadership) and structural (e.g., modification of work processes) [13].

### Outcomes

The primary outcome is the self-assessed physical and mental work ability of employees. The secondary outcome is clients’ satisfaction with care. In addition, since the intervention effects are assumed to be the consequence of the reduction of the psychosocial load at work, the effort-reward imbalance of workers will be considered in additional analyses in order to evaluate a potential mechanism by which the intervention may have an effect on work ability [3]. These analyses will rely on theoretical considerations of the work ability concept in which the health status of individual workers is believed to be a determining antecedent of the appraisal of one’s own work ability [14]. All outcomes are measured by appropriate validated psychometric instruments freely available to scientists for research purposes. The questionnaires for healthcare workers comprise basic socio-demographic information, physical and psychosocial working conditions, work ability, and perceived physical and mental health (Table S1, Supplementary material 1). The questionnaires for clients include questions related to basic socio-demographic information, a set of scales measuring satisfaction with care, and a generic general health question (Tables S2 and S3, Supplementary material 1). Healthcare workers may fill out the questionnaires either online or as a paper-pencil version, and receive on request from the authors at Ulm University a short personalised report of their responses to the survey. The questionnaires for clients are available as a paper-pencil version only.

### Sample size

The sample size was calculated with the formulae provided by Dreyhaupt et al. (2017) [15], which considers the intraclass correlation coefficient (ICC), the average cluster size (m), the number of clusters (J), and the design effect (DE). The approach for calculating the sample size was to estimate the minimum effect attainable for a given sample and cluster size. An estimate of the ICC = 0.037 was taken from the intervention study of Mongini et al. (2012) conducted with a sample of Italian public servants [16]. Furthermore, it was assumed that a total of 50 clusters could be expected with an average cluster size of m = 5. Under these assumptions, a design effect DE = 1 + ICC*(m-1) = 1.33 was estimated, so that a total sample size of 500 participants was found to be required in order to detect a minimum effect of 0.30 at the 80% power and 5% significance levels. A total sample size of 500 individuals in a cluster-randomised design corresponds to an effective total sample size of 375 in a study with individual randomisation [15].

### Randomisation and implementation

**Fig. 2:**
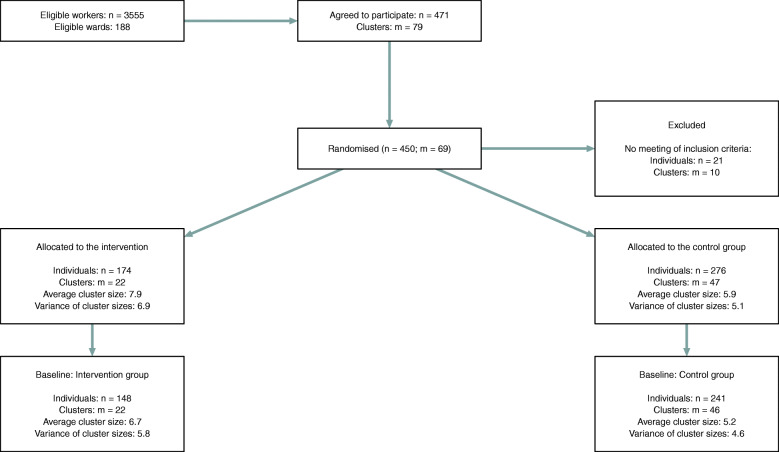
Flow diagram of healthcare workers (n) and clusters (m) from recruitment to baseline analysis

Healthcare workers registered for the surveys by filling out a registration form addressed to Ulm University including personal information and the name of the ward they usually work in. Wards were then aggregated by the authors at Ulm University in clusters as described above, and stratified by hospital and elderly care ward. A list containing hospitals, clusters and number of registered participants was provided by the first author to the Institute of Epidemiology and Medical Biometry at Ulm University which generated the random allocation sequence and assigned clusters to interventions for the hospitals, independently from the authors at Ulm University and the consultants providing the intervention. The randomisation of clusters in the hospitals was performed in two steps with the statistical environment R. In the first step, the probability of being assigned to the intervention group was proportional to cluster size in each hospital, and a total of 10 clusters (i.e., one intervention cluster per hospital) were allocated to the intervention arm. Given the large variation of cluster sizes (Fig. 2), a sampling schedule proportional to size was required in the first step in order to ensure the generalisability of results by including the largest clusters in the hospitals, and to counter the expected power loss due to cluster and individual sample attrition in the subsequent follow-up measurements. In the second step, the random allocation proceeded by simple random sampling. However, given that only four consultants provide the intervention, a 1:1 allocation scheme for the remaining clusters was not feasible due to personnel limitations. Thus, the number of additional clusters in the intervention group in the hospitals was limited to 10. On the other hand, since the elderly care centre comprises only four wards, a simple random allocation in proportion 1:1 was performed in that case by the first author at Ulm University.

### Allocation concealment mechanism

Since the intervention targets whole wards, a complete blinding of participants and consultants in this study is not feasible. All healthcare workers in the intervention clusters, and probably also those in the control clusters, are aware of the allocation to the intervention and control arm, respectively. The consultants delivering the intervention know which clusters were allocated to the intervention arm, but are unaware of which clusters belong to the control arm in the hospitals. In order to reduce the risk of bias resulting from workers being aware to be either in the intervention or control arm, the baseline measurement was conducted before cluster randomisation and implementation of the interventions. In addition, the identification of clusters and the recruitment of participants took place prior to randomisation. Hence, neither the investigators at Ulm University nor the consultants had foreknowledge of the allocation results at the time of participant recruiting and cluster identification. Furthermore, the risk of contamination between intervention and control clusters in the hospitals and elderly care wards was reduced by the clustering of wards according to the specialisation area and the organisational structure of the participating organisations (e.g., separate building areas, different buildings or hospitals). Even though about 6% of participating workers reported working frequently in more than one ward, these workers actually shift between wards belonging to single clusters (e.g., wards within the cluster cardiology), so that there is practically no risk of contamination between the intervention and control arm by the time of random allocation. Survey data collection and analysis, and the process evaluation of the implementation are performed by the authors at Ulm University, independently from both the consultants responsible for delivering the interventions, and the participating healthcare providers. At the end of the study, voluntary wards in the control arm will be given the opportunity to implement the intervention.

### Statistical methods

The intervention effects on employees’ work ability and clients’ satisfaction with care will be estimated by means of generalised linear mixed-effect (GLM) regression models [17] with two levels of nesting (clusters within healthcare organisations). The GLM models are appropriate for cluster-randomised trials, since they account for the clustered structure of data in a longitudinal design [18]. Missing data at the end of the study will be handled by imputation routines and sensitivity analyses [19, 20]. The mean scores of the psychometric scales will be computed with available items if no more than 30% of the items defining the scale are missing [21]. The ICC at baseline are estimated from the variance components of a random-intercept model with two levels of nesting (clusters within organisations). The baseline data are analysed with a series of GLM Bayesian regression models (also called hierarchical models) by means of Markov Chain algorithms as described elsewhere [22]. The goodness-of-fit was assessed by the Deviance Information Criterion (DIC). Lower values of the DIC statistic indicate a better model fit [23]. Given the role of the effort-reward imbalance as a potential mediating mechanism between the intervention and the main outcomes (see methods section), the associations between physical and mental work ability with effort-reward imbalance and overcommitment are investigated in three regression models with the workers dataset. Due to the fact that health in the work ability concept is thought to be a determining antecedent of one’s own work ability perceptions, the fully adjusted models include two SF12-equivalent physical and mental health component scores [24]. In addition, for the patients dataset, the associations between the four indicators of satisfaction with care and the cluster-levels of psychosocial load of healthcare workers are estimated.

### Process evaluation

Process evaluations are highly valuable for understanding how discrepancies between the expected and observed outcomes may be related to context influences and implementation issues arising in complex interventions [6]. In the present study, the process evaluation of both implementation issues and context is based on the approach suggested by Linnan and Steckler [25], in which special consideration is put to the degree of receptivity and engagement of the workers to the intervention. Moreover, since previous research has shown that employees are more likely to participate in the activities of interventions, if they believe they can influence the intervention contents [26], group-related processes associated with the so-called collective self-efficacy [27] will also be taken into account. It is hypothesised that workers will be more engaged in the intervention, if they believe the group is capable of achieving the intervention goals (i.e., high collective self-efficacy). The evaluation will be based on information collected in questionnaires which were developed specifically for this intervention on the basis of previous literature reviews on process evaluation [28–30]. The questionnaires collect information on the assessment of the study participants on several process variables which have been identified in the pertinent literature as decisive for the attainment of intervention goals such as perceived support by management, conflict and collaboration in workshop groups, the expected personal benefit from the intervention, and the feasibility of intervention activities. Furthermore, according to those literature reviews, support from key stakeholders such as managers and supervisors may have a substantial impact on the intervention outcomes. Since most members of the initiatives circles described above have a leading position, specific questionnaires will be developed and deployed among the members of those circles. The adequacy of the newly developed questionnaires will be investigated in a pretest phase including cognitive Interviews and psychometric analyses.

## Results

The flow diagram of the number of individual participants and clusters from recruitment to baseline is provided in Fig. 2. Total response rates for workers and patients at baseline were about 13 and 53%, respectively. The socio-demographic characteristics of workers and clients, and the descriptive statistics of the main outcomes in both the intervention and control groups are reported in Tables 1 and 2. In the 11 health organisations participating in the intervention, total of 67 clusters were defined covering about 68% of all wards and including 24 health services areas such as anaesthesia, intensive care units, geriatrics, psychiatry, surgery, cardiology, paediatrics, urology, trauma surgery and emergency. Since most participating organisations are general hospitals providing a similar range of health services, the specific characteristics of clusters across organisations are rather balanced: It was found that for 57 clusters there were at least two clusters of a similar health service type in two different hospitals. For instance, for two intensive care clusters in two different hospitals, one intensive care cluster in one hospital was assigned to the intervention group, and the other cluster to the control group.

**Table 1:** Descriptive statistics of the healthcare workers datasets. Percent values for categorical variables, means and standard deviation in parentheses for continuous variables. Missing values per variable or scale

Variable	Control	Intervention	Missing
**Healthcare workers (*n*** **= 386)**			
*Age*			1
Age 18–39	23.1	27.2	
Age 40–54	52.5	46.3	
Age 55 and older	24.4	26.5	
*Sex*			5
Male	23.0	16.4	
Female	77.0	83.6	
*Psychometric scales*			
Physical work ability	3.15 (0.98)	3.10 (0.91)	4
Mental work ability	3.05 (0.94)	3.04 (0.87)	6
Cognitive demands	4.29 (0.46)	4.32 (0.41)	1
Emotional demands	3.81 (0.62)	3.85 (0.57)	2
Low job control	3.24 (0.65)	3.14 (0.67)	1
Low predictability of work tasks	2.87 (0.70)	2.83 (0.73)	1
Role clarity	2.16 (0.62)	2.11 (0.70)	2
Role conflict	3.12 (0.75)	3.19 (0.77)	1
Low development chances	2.30 (0.62)	2.20 (0.63)	1
Efforts	2.62 (0.69)	2.58 (0.61)	4
Rewards	1.84 (0.59)	1.82 (0.53)	23
Effort-Reward Imbalance	0.89 (0.40)	0.85 (0.32)	26
Overcommitment	2.59 (0.61)	2.59 (0.56)	2
Supervisor behaviours	3.16 (0.94)	3.16 (0.95)	3
Unsupportive colleagues	1.88 (0.67)	1.94 (0.74)	2
Negative affect	1.86 (0.64)	1.77 (0.55)	3

**Table 2:** Descriptive statistics of the clients datasets. Percent values for categorical variables, means and standard deviation in parentheses for continuous variables. Missing values per variable or scale

Variable	Control	Intervention	Missing
**Patients (*n*** **= 632)**			
*Age*			4
Age 18–49	22.9	17.5	
Age 50–69	38.4	27.9	
Age 70 and older	38.7	54.6	
*Sex*			2
Male	47.1	50.0	
Female	52.9	50.0	
Length of hospital stay (days)	7.26 (7.84)	6.90 (7.61)	5
*Psychometric scales*			
Trust	3.71 (0.46)	3.77 (0.38)	4
Support	3.58 (0.57)	3.68 (0.46)	28
Availability	3.57 (0.51)	3.64 (0.44)	2
Decisional control	4.36 (0.56)	4.35 (0.51)	4
**Individuals in elderly care (*n*** **= 150)**			
*Age*			0
Age 50–79	42.9	24.2	
Age 80–89	50.0	49.5	
Age 90 and older	7.1	26.3	
*Sex*			0
Male	42.9	28.4	
Female	57.1	71.6	
Years being in elderly care	4.54 (3.74)	3.96 (3.78)	8
*Psychometric scales*			
Trust	3.29 (0.72)	3.76 (0.43)	7
Support	3.07 (0.82)	3.60 (0.45)	9
Decisional control	4.10 (0.89)	4.47 (0.67)	12
Person-focused care	3.19 (1.04)	3.30 (1.20)	10

The estimates of the effective sample size (N_eff_) and the ICC corresponding to the workers’ physical and mental work ability were N_eff_ = 345 and 423, and ICC = 0.05 and 0.01, respectively. The average cluster size in the workers sample was m = 6.53, which yields design effect estimates of DE = 1.29 and 1.05 for the main outcomes. Thus, it will be possible to estimate effect sizes of at least 0.30 and 0.27 at the 80% power and 5% significance levels, for the workers’ physical and mental work ability, respectively. The estimates for the patients dataset were N_eff_ = 234, average ICC = 0.09, m = 19.5, and DE = 2.7. Since data of approximately 1200 additional patients will be collected cross-sectionally at the T1 and T2 follow-ups, a final effective sample size of approximately 234*3 = 702, and an average effect size of about 0.21 concerning patients satisfaction with care may be estimated at the 80% power and 5% significance levels. Finally, the power analysis for the elderly care dataset yielded an average ICC = 0.17, m = 30.7, and DE = 6.32, and an effective sample size of 24 per survey. Thus, for this dataset, at the end of the study, an effect size of about 0.66 can be estimated at the 80% power and 5% significance levels with a final effective sample size N_eff_ = 74 of elderly care clients.

The results of the statistical analyses at baseline are reported in Tables 3 and 4 for workers and clients, respectively. There were no baseline differences between the intervention and control groups in the datasets regarding the main outcomes of the study. Among workers, it was found that higher scores of effort-reward imbalance and overcommitment are associated with lower scores of physical and mental work ability (Table 3). These associations seem to be partly mediated by the levels of negative affect, and physical and mental health perceptions, as indicated by the lower magnitude of the regression coefficients in the fully adjusted models (models 2 and 3 in Table 3, respectively). Among patients, the results did not suggest any association between the perceived effort-reward imbalance and overcommitment of healthcare workers at the cluster level and patients’ satisfaction with care (Table 4).

**Table 3:** Bayesian linear mixed models for the healthcare workers dataset (complete cases). Dependent variables: physical and mental work ability. Model 1 is adjusted for age, gender, and intensive care unit vs. other wards. Model 2 adjusts also for negative affect and organisation type (general hospital vs. getriatric and psychiatric hospitals). Model 3 is the fully adjusted model with physical and mental health component scores. Beta: regression coefficient, SE: standard error, and 95% CI: confidence intervals at the 95% level. ERI: effort-reward imbalance. DIC: deviance information criterion. *N* = 346

Variable	Physical work ability						Mental work ability					
	Model 1		Model 2		Model 3		Model 1		Model 2		Model 3	
	Beta (SE)	95% CI	Beta (SE)	95% CI	Beta (SE)	95% CI	Beta (SE)	95% CI	Beta (SE)	95% CI	Beta (SE)	95% CI
Intercept	4.79 (0.28)	[4.20; 5.29]	5.00 (0.30)	[4.40; 5.59]	−0.18 (0.72)	[−1.61; 1.12]	4.90 (0.27)	[4.38; 5.42]	5.51 (0.28)	[4.90; 6.00]	2.35 (0.68)	[1.07; 3.78]
Intervention (ref. control)	− 0.10 (0.11)	[− 0.32; 0.10]	− 0.09 (0.11)	[− 0.32; 0.12]	− 0.06 (0.11)	[− 0.28; 0.14]	− 0.05 (0.11)	[− 0.26; 0.16]	− 0.09 (0.11)	[− 0.31; 0.10]	− 0.04 (0.11)	[− 0.27; 0.15]
ERI	− 0.80 (0.17)	[−1.13; − 0.50]	− 0.59 (0.18)	[− 0.92; − 0.24]	− 0.24 (0.18)	[− 0.58; 0.09]	− 0.42 (0.17)	[− 0.74; − 0.10]	−0.12 (0.19)	[− 0.50; 0.24]	0.01 (0.18)	[− 0.33; 0.37]
Overcommitment	−0.28 (0.11)	[− 0.50; − 0.09]	− 0.15 (0.12)	[− 0.38; 0.07]	− 0.04 (0.11)	[− 0.26; 0.18]	−0.56 (0.11)	[− 0.76; − 0.35]	−0.38 (0.11)	[− 0.62; − 0.19]	−0.25 (0.11)	[− 0.47; − 0.05]
DIC	881		874		773		863		827		793	
Residual variance	0.884		0.874		0.783		0.866		0.831		0.800

**Table 4:** Bayesian linear mixed models for the clients datasets (complete cases). The models for the patients dataset are adjusted for age, gender, organisation type (general hospital vs. getriatric and psychiatric hospitals), general health, and education. The models for the elderly care dataset are adjusted for age, gender, general health, and education. Beta: regression coefficient, SE: standard error, and 95% CI: confidence intervals at the 95% level. DIC: deviance information criterion. Effort-reward imbalance (ERI) and overcommitment values correspond to the average of psychosocial load of workers at the cluster level

**Patients (*n*** **= 577)**								
**Dependent variables:**	**Trust in carers**		**Support by carers**		**Availability of carers**		**Decisional control over care**	
	**Beta (SE)**	**95% CI**	**Beta (SE)**	**95% CI**	**Beta (SE)**	**95% CI**	**Beta (SE)**	**95% CI**
Intercept	3.44 (0.65)	[2.16; 4.83]	3.35 (0.69)	[2.16; 4.82]	3.59 (0.64)	[2.42; 4.95]	4.54 (0.68)	[3.35; 5.94]
Intervention (ref. control)	0.03 (0.09)	[−0.16; 0.22]	0.06 (0.09)	[−0.11; 0.23]	0.04 (0.09)	[−0.16; 0.19]	− 0.01 (0.09)	[− 0.20; 0.16]
Physical work ability	0.00 (0.07)	[−0.14; 0.13]	0.01 (0.07)	[−0.13; 0.13]	0.01 (0.07)	[−0.15; 0.13]	0.02 (0.07)	[−0.13; 0.16]
Mental work ability	−0.00 (0.09)	[−0.17; 0.20]	0.01 (0.10)	[−0.17; 0.20]	− 0.05 (0.09)	[− 0.23; 0.13]	−0.07 (0.10)	[− 0.26; 0.10]
ERI	0.01 (0.25)	[−0.48; 0.47]	0.11 (0.25)	[−0.34; 0.64]	0.04 (0.26)	[−0.43; 0.56]	0.32 (0.26)	[−0.26; 0.79]
Overcommitment	−0.01 (0.18)	[−0.36; 0.35]	− 0.11 (0.19)	[− 0.43; 0.28]	−0.09 (0.18)	[− 0.43; 0.29]	−0.22 (0.19)	[− 0.57; 0.17]
DIC	902		1044		985		1067	
Residual variance	0.550		0.607		0.583		0.617	
**Individuals in elderly care (*n*** **= 96)**								
**Dependent variables:**	**Trust in carers**		**Support by carers**		**Decisional control over care**		**Person-focused care**	
	**Beta (SE)**	**95% CI**	**Beta (SE)**	**95% CI**	**Beta (SE)**	**95% CI**	**Beta (SE)**	**95% CI**
Intercept	2.96 (0.56)	[1.76; 3.97]	3.09 (0.59)	[1.98; 4.30]	4.19 (0.57)	[3.13; 5.31]	3.69 (0.60)	[2.48; 4.83]
General health	0.13 (0.12)	[−0.13; 0.35]	0.08 (0.12)	[−0.15; 0.33]	−0.05 (0.12)	[−0.28; 0.19]	0.05 (0.13)	[−0.20; 0.29]
Intervention (ref. control)	0.38 (0.38)	[−0.31; 1.13]	0.39 (0.39)	[−0.39; 1.12]	0.22 (0.39)	[−0.50; 1.01]	0.16 (0.38)	[−0.57; 0.86]
DIC	212		207		221		292	
Residual variance	0.899		0.890		0.908		1.018	

## Discussion

The results at baseline suggest that the random allocation of clusters was satisfactory, since no substantial differences were observed between the intervention and control arm regarding the primary outcomes. According to the power analyses reported in the results section, one strength of the intervention is that effect sizes of at least 0.30 and 0.27 can be estimated at the 80% power and 5% significance levels, for physical and mental work ability of workers, respectively. Given that previous randomised organisational interventions have reported even larger effect sizes (standardised mean differences of − 1.23, − 0.55 and − 0.35) [7], the present study has sufficient power to detect substantial changes in the main outcomes.

A further strength of the study concerns the indication of an acceptable internal validity. Mainly successfully and widely tested questionnaires showing satisfying to good psychometric properties in previous studies [24, 31–35] and the present intervention as well (average Cronbach’s alpha over all instruments 0.74, see Supplementary material 1), were applied to measure independent (e.g., workload) and dependent variables (e.g., work ability, clients’ satisfaction). These measures allow comparisons of the present results with findings from other studies. Concerning the first study hypothesis, the results show associations of measures of effort-reward imbalance with work ability in workers. Hence, it is plausible to expect that a reduction of workload may result in increases of perceived work ability. In addition, the results indicate that these associations are mediated by perceived health (model 3 in Table 3). This mediation has been hypothesised previously, but has not been shown empirically so far [36]. However, the second hypothesis stating that the reduction of workload increases patients satisfaction did not receive support, since no cross-sectional associations were observed between workload and patients satisfaction (Table 4). Due to the low number of clusters in the elderly care centre, the second hypothesis cannot be investigated with the elderly care dataset.

Besides the consideration of the primary and secondary outcomes described in the methods section, by the end of the study further analyses may be performed in order to investigate how the intervention measures are related to specific working conditions. However, as far as the intervention measures are proposed by the workers themselves, the feasibility and scope of such additional statistical analyses will depend on the type and number of measures being actually implemented, the type of psychosocial factors potentially targeted by the interventions, and the extent to which specific intervention targets may be aggregated across clusters as individual, inter-personal and structural measures (see methods section). At the same time, the results obtained from the process evaluation will be used to inform the interpretation of results by focusing, among others, on the commitment of stakeholders, management and workers, and the potential role of collective self-efficacy expectations.

Because the overall participation rate in the intervention was rather low (13%), some basic socio-demographic statistics of the sample were compared to the corresponding values of the population of carers obtained from available hospital records. It was found that the participants are on average older than the whole population of hospital carers (46 vs. 43 years old), have a longer working experience (20 years vs. 14 years), and the proportion of males in the sample is slightly larger (17% vs. 22%). In view of these differences, the results of the present intervention should be interpreted with some caution regarding younger carers with less work experience.

Although the low participation rate at the individual level is an important limitation of the present study, several observations indicate that (1) participating workers are representative of the eligible healthcare workers, (2) the intervention measures are tailored to the workers’ needs and, (3) consequently, they may be effective at improving the working conditions in the wards. First, the number of wards included in the intervention account for about 68% of all hospital wards. Hence, even though the participation rate at the individual level is low (13%), the ward coverage is high (68%). Second, as stated in the methods section, the intervention is performed at the ward level, and, hence, the number of workers taking part in, and receiving, the intervention is actually higher than the number of workers filling out the questionnaires. Thus, the intervention measures are expected to address the most relevant issues for all workers in the intervention wards. Finally, the intervention measures are proposed and prioritised by the workers themselves during the interviews and workshops. Consequently, it is likely that these measures are effective at improving the working conditions in the intervention wards.

## Conclusions

In conclusion, the results suggest that the implementation of the study design has been satisfactory so far. The intervention is expected to provide evidence of relatively small to medium-size effects of the intervention activities on the work ability of healthcare workers and on clients’ satisfaction with care.

## Supplementary information

**Additional file 1: Supplementary file 1.** Additional descriptive statistics.

**Additional file 2: Supplementary file 2.** CONSORT check-list for cluster-randomised trials.

## Data Availability

Due to data protection regulations there is no permission to make the datasets available to third parties.

## Abbreviations

CI: Confidence interval
DE: Design effect
DIC: Deviance Information Criterion
ERI: Effort-reward imbalance
GLM: Generalised linear-mixed
ICC: Intraclass correlation coefficient
M: Average cluster size
N_eff_: Effective sample size
