# Supplementary material for: Working conditions of healthcare workers and clients’ satisfaction with care: study protocol and baseline results of a cluster-randomised workplace intervention
Source: BMC Public Health. 2020 Aug 25;20:1281. doi: 10.1186/s12889-020-09290-4 (PMC7449031; doi:10.1186/s12889-020-09290-4)
Supplement: Supplementary file 1 — Additional file 1: Supplementary file 1. Additional descriptive statistics. [file 12889_2020_9290_MOESM1_ESM.pdf]

Title: Working conditions of healthcare workers and clients' satisfaction with care: a cluster-randomised workplace intervention

Supplementary material 1

In this supplementary file, the descriptive statistics of the scales used in the questionnaires are provided.

Table S1. Descriptive statistics of the instruments used in the healthcare workers surveys. SD: standard deviation, Alpha: Cronbach's alpha, n.a: not applicable. N = 386.

Instruments	Mean	SD	Alpha
**Work Ability Index (WAI)**[1] (1)			
Physical work ability	3.16	0.95	n.a.
Mental work ability	3.03	0.92	n.a.
**Copenhagen Psychosocial Questionnaire (COPSOQ)**[2] (2)			
Quantitative demands	4.29	0.46	0.68
Emotional demands	3.82	0.61	0.73
Influence at work	3.20	0.67	0.68
Predictability	2.86	0.71	0.59
Role clarity	2.17	0.67	0.76
Role conflicts	3.15	0.76	0.74
Possibilites for development	2.27	0.65	0.59
Intention to leave	1.98	0.89	0.75
Social community at work	3.22	0.71	0.64
Quality of leadership	3.15	0.94	0.92
Social support from colleagues	1.93	0.70	0.85
Work family conflict	2.64	0.87	0.83
**Activity and Work Analysis in Hospitals (TAA-KH-S)**[3] (3)			
Patient-related demands	2.59	0.30	0.71
**Effort-Reward-Imbalance (ERI)**[4] (4)			
Effort	2.59	0.67	0.80
Reward	1.83	0.56	0.79
Overcommitment	2.60	0.59	0.57
**German Version of the Positive and Negative Affect Scale (PANAS)**[5] (5)			
Negative affect	1.84	0.62	0.86
**Health related Quality of Life – SF 12 (Version of the German Socio-Economic Panel)**[6] (6)			
Physical functioning	69.90	29.36	n.a.

Instruments	Mean	SD	Alpha
Role physical	66.51	24.78	n.a.
Bodily pain	65.70	28.62	n.a.
Social functioning	67.57	27.24	n.a.
Role emotion	69.82	23.29	n.a.
Vitality	49.63	21.71	n.a.
Mental Health	56.68	20.40	n.a.
General Health	60.47	23.27	n.a.

[1] The WAI assesses the capacity of healthcare workers to manage demands of the psychosocial environment to meet their individual needs of wellbeing, given their mental resources and physical health.
[2] The COPSOQ questionnaire encompasses a broad range of psychosocial work characteristics. The COPSOQ is based on various leading concepts and theories in occupational health.
[3] The TAA-KH-S assesses psychosocial work characteristics of healthcare workers.
[4] The ERI-Model states that a lack in social reciprocity (represented by high efforts and low rewards) can lead to emotional distress and, in consequence, to adverse effects on health.
[5] The PANAS is used to measure mood states in relation to the dimensions positive and negative affect.
[6] The Health-related quality of life (HRQOL) measures an individual's perceived physical and mental health.

Table S2. Descriptive statistics of the instruments used in the patients surveys. SD: standard deviation, Alpha: Cronbach's alpha, n.a: not applicable. N = 632.

Instruments	Mean	SD	Alpha
**Cologne Patient Questionnaire (KPF)**[1] (7)			
Trust in carers	3.73	0.43	0.86
Support by carers	3.63	0.53	0.85
Availability of information and support by carers	3.61	0.50	0.81
**Health related Quality of Life – SF 12 (Version of the German Socio-Economic Panel)**			
General health	2.91	0.99	n.a.
**Individualized Care Scale (ICS)**[2] (8)			
Patients decisional control over care	4.35	0.54	0.66

[1] The KPF measures the patient's satisfaction with the quality of care.
[2] The ICS asseses the degree of perceived patient-centred care, and support about decision-making processes about their care.

Table S3. Descriptive statistics of the instruments used in the surveys of individuals in elderly care. SD: standard deviation, Alpha: Cronbach's alpha, n.a: not applicable. N = 150.

Instruments	Mean	SD	Alpha
**Cologne Patient Questionnaire (KPF)**[1]			
Trust in carers	3.61	0.57	0.82
Support by carers	3.46	0.62	0.76
Availability of information and support by carers	3.48	0.51	0.42
**Health related Quality of Life – SF 12 (Version of the German Socio-Economic Panel)**			
General health	2.80	0.86	n.a.

Instruments	Mean	SD	Alpha
**Individualized Care Scale (ICS)**[2]			
Patients decisional control over care	4.38	0.73	0.76
**German Version of the Resident Quality of Life Questionnaire**[3] (9)			
Person-focused care	3.30	1.13	0.85

[1] In order to apply the KPF scales to the elderly care, some items were adapted to the appropriate context, and not applicable items were excluded.
[2] In order to apply the ICS scales to the elderly care, some items were adapted to the appropriate context, and not applicable items were excluded.
[3] The Resident Quality of Life Questionnaire measures the quality of life of individuals in elderly nursing homes. In the present study only three items of the scale “person-focused care” were included.

References

[1] Hasselhorn H-M, Freude G. Der Work Ability Index – ein Leitfaden. Dortmund: Bundesanstalt für Arbeitsschutz und Arbeitsmedizin; 2007.

[2] Nübling M, Stößel U, Hasselhorn H, Michaelis M, Hofmann F. Methoden zur Erfassung psychischer Belastungen. Erprobung eines Messinstruments (COPSOQ). Dortmund: Bundesanstalt für Arbeitsschutz und Arbeitsmedizin; 2005.

[3] Büssing A, Glaser J. Das Tätigkeits- und Arbeitsanalyseverfahren für das Krankenhaus - Selbstbeobchtungsversion (TAA-KH-S). Göttingen: Hogrefe; 2002.

[4] Siegrist J, Starke D, Chandola T, Godin I, Marmot M, Niedhammer I et al.. The measurement of Effort-Reward Imbalance at work: European comparisons. Social Science & Medicine. 2004;58(8):1483-1499.

[5] Breyer B, Bluemke M. Deutsche Version der Positive and Negative Affect Schedule PANAS (GESIS Panel). Mannheim: gesis; 2016.

[6] Nübling M, Andersen H, Mühlbacher A. Entwicklung eines Verfahrens zur Berechnung der körperlichen und psychischen Summenskalen auf Basis der SOEP – Version des SF 12 (Algorithmus). Berlin: DIW Berlin; 2006.

[7] Pfaff H, Nitzsche A, Scheibler F, Steffen P. Der Kölner Patientenfragebogen für Brustkrebs (KPF-BK). Köln: Universität zu Köln; 2009.

[8] Köberich S, Suhonen R, Feuchtinger J, Farin E. The German version of the Individualized Care Scale - assessing validity and reliability. Patient Preference and Adherence. 2015;:483. https://doi.org/10.2147/ppa.s77486.

[9] Sommerhalder K, Gugler E, Conca A, Bernet M, Bernet N, Serdaly C et al.. Lebens- und Pflegequalität im Pflegeheim – Beschreibende Ergebnisse der Befragung von Bewohnerinnen und Bewohnern in Pflegeheimen in der Schweiz. Residents’ Perspectives of Living in Nursing Homes in Switzerland (RESPONS). Bern: Berner Fachhochschule; 2015.
