# Supplementary material for: Working conditions of healthcare workers and clients’ satisfaction with care: study protocol and baseline results of a cluster-randomised workplace intervention
Source: BMC Public Health. 2020 Aug 25;20:1281. doi: 10.1186/s12889-020-09290-4 (PMC7449031; doi:10.1186/s12889-020-09290-4)
Supplement: Supplementary file 2 — Additional file 2: Supplementary file 2. CONSORT check-list for cluster-randomised trials. [file 12889_2020_9290_MOESM2_ESM.pdf]

Title: Working conditions of healthcare workers and clients' satisfaction with care: a cluster-randomised workplace intervention

Supplementary file 2

In this supplementary file, the CONSORT checklist for cluster-randomised trials is provided.

Table S1. CONSORT 2010 checklist of information to include when reporting a cluster randomised trial (1).

Section/Topic	Item No	Standard Checklist item	Extension for cluster designs	Page number and section
**Title and abstract**				
	1a	Identification as a randomised trial in the title	Identification as a cluster randomised trial in the title	Introduction, p.1
	1b	Structured summary of trial design, methods, results, and conclusions (for specific guidance see CONSORT for abstracts)		Abstract, p. 2
**Introduction**				
**Background and objectives**	2a	Scientific background and explanation of rationale	Rationale for using a cluster design	Study design, p. 4
	2b	Specific objectives or hypotheses	Whether objectives pertain to the the cluster level, the individual participant level or both	Background, p. 3
**Methods**				
**Trial design**	3a	Description of trial design (such as parallel, factorial) including allocation ratio	Definition of cluster and description of how the design features apply to the clusters	Participants, p. 5;Randomisation and implementation, p. 8-9
	3b	Important changes to methods after trial commencement (such as eligibility criteria), with reasons		Not applicable
**Participants**	4a	Eligibility criteria for participants	Eligibility criteria for clusters	Participants, p. 4-5
	4b	Settings and locations where the data were collected		Outcomes, p. 7-8
**Interventions**	5	The interventions for each group with sufficient details to allow replication, including how and when they were actually administered	Whether interventions pertain to the cluster level, the individual participant level or both	The intervention, p. 5-7
**Outcomes**	6a	Completely defined pre-specified primary and secondary outcome measures, including how and when they were assessed	Whether outcome measures pertain to the cluster level, the individual participant level or both	Study design, p.4;Participants, p. 4-5; Outcomes, p. 7-8

Section/Topic	Item No	Standard Checklist item	Extension for cluster designs	Page number and section
	6b	Any changes to trial outcomes after the trial commenced, with reasons		Not applicable
**Sample size**	7a	How sample size was determined	Method of calculation, number of clusters(s) (and whether equal or unequal cluster sizes are assumed), cluster size, a coefficient of intracluster correlation (ICC or *k*), and an indication of its uncertainty	Sample size, p. 8
	7b	When applicable, explanation of any interim analyses and stopping guidelines		Not applicable
**Randomisation:**				
**Sequence generation**	8a	Method used to generate the random allocation sequence		Randomisation and implementation, p. 8-9
	8b	Type of randomisation; details of any restriction (such as blocking and block size)	Details of stratification or matching if used	Randomisation and implementation, p. 8-9
**Allocation concealment mechanism**	9	Mechanism used to implement the random allocation sequence (such as sequentially numbered containers), describing any steps taken to conceal the sequence until interventions were assigned	Specification that allocation was based on clusters rather than individuals and whether allocation concealment (if any) was at the cluster level, the individual participant level or both	Allocation concealment mechanism, p. 9-10
**Implementation**	10	Who generated the random allocation sequence, who enrolled participants, and who assigned participants to interventions	Replace by 10a, 10b and 10c	Randomisation and implementation, p. 8-9
	10a		Who generated the random allocation sequence, who enrolled clusters, and who assigned clusters to interventions	Randomisation and implementation, p. 8-9
	10b		Mechanism by which individual participants were included in clusters for the purposes of the trial (such as complete enumeration, random sampling)	Randomisation and implementation, p. 8-9
	10c		From whom consent was sought (representatives of the cluster, or individual cluster members, or both), and whether consent was sought before or after randomisation	Participants, p. 4-5
**Blinding**	11a	If done, who was blinded after assignment to interventions (for example, participants, care providers, those assess-		Not applicable; complete blinding is not feasible in this study

Section/Topic	Item No	Standard Checklist item	Extension for cluster designs	Page number and section
		ing outcomes) and how		
	11b	If relevant, description of the similarity of interventions		Not applicable
**Statistical methods**	12a	Statistical methods used to compare groups for primary and secondary outcomes	How clustering was taken into account	Statistical methods, p. 10-11
	12b	Methods for additional analyses, such as subgroup analyses and adjusted analyses		Statistical methods, p. 10-11
**Results**				
**Participant flow (a diagram is strongly recommended)**	13a	For each group, the numbers of participants who were randomly assigned, received intended treatment, and were analysed for the primary outcome	For each group, the numbers of clusters that were randomly assigned, received intended treatment, and were analysed for the primary outcome	Results, p. 11-12; Figure 1: Flow diagram
	13b	For each group, losses and exclusions after randomisation, together with reasons	For each group, losses and exclusions for both clusters and individual cluster members	Results, p. 11-12; Figure 1: Flow diagram
**Recruitment**	14a	Dates defining the periods of recruitment and follow-up		Study design, p. 4
	14b	Why the trial ended or was stopped		Not applicable
**Baseline data**	15	A table showing baseline demographic and clinical characteristics for each group	Baseline characteristics for the individual and cluster levels as applicable for each group	Results, p. 13, Table 1
**Numbers analysed**	16	For each group, number of participants (denominator) included in each analysis and whether the analysis was by original assigned groups	For each group, number of clusters included in each analysis	Results, p. 12, Figure 1
**Outcomes and estimation**	17a	For each primary and secondary outcome, results for each group, and the estimated effect size and its precision (such as 95% confidence interval)	Results at the individual or cluster level as applicable and a coefficient of intracluster correlation (ICC or k) for each primary outcome	Results, p. 12
	17b	For binary outcomes, presentation of both absolute and relative effect sizes is recommended		Not applicable
**Ancillary analyses**	18	Results of any other analyses performed, including subgroup analyses and adjusted analyses, distinguishing pre-specified from exploratory		Results, p. 15-16, Tables 2 and 3
**Harms**	19	All important harms or unintended effects in each group (for specific guidance see CONSORT for harms)		Not applicable

Section/Topic	Item No	Standard Checklist item	Extension for cluster designs	Page number and section
**Discussion**				
**Limitations**	20	Trial limitations, addressing sources of potential bias, im-precision, and, if relevant, multiplicity of analyses		Discussion, p. 18
**Generalisability**	21	Generalisability (external validity, applicability) of the trial findings	Generalisability to clusters and/or individual participants (as relevant)	Discussion, p. 18
**Interpretation**	22	Interpretation consistent with results, balancing benefits and harms, and considering other relevant evidence		Discussion, p. 17-18
**Other information**				
**Registration**	23	Registration number and name of trial registry		Abstract, p. 3
**Protocol**	24	Where the full trial protocol can be accessed, if available		Not applicable
**Funding**	25	Sources of funding and other support (such as supply of drugs), role of funders		Funding, p. 19

References

[1] Campbell MK, Piaggio G, Elbourne DR, Altman DG. Consort 2010 statement: extension to cluster randomised trials. BMJ. 2012;345 (sep04 1):e5661-e5661. https://doi.org/10.1136/bmj.e5661.
